# Glycemic Control in Children and Adolescents with Type 1 Diabetes: From Challenges to Innovation

**DOI:** 10.3390/children12060708

**Published:** 2025-05-29

**Authors:** Stefano Passanisi, Agata Chobot, Claudia Piona

**Affiliations:** 1 Department of Human Pathology in Adult and Developmental Age “Gaetano Barresi”, University of Messina, 98122 Messina, Italy; 2 Department of Pediatrics, Institute of Medical Sciences, University of Opole, 45-040 Opole, Poland; agata.chobot@gmail.com; 3 Pediatric Diabetes and Metabolic Disorders Unit, Regional Center for Pediatric Diabetes, University City Hospital, 37126 Verona, Italy; claudia.piona@univr.it

Over the past two decades, the landscape of type 1 diabetes (T1D) management in pediatric populations has undergone a remarkable transformation. The introduction and evolution of advanced technologies, including real-time and intermittently scanned continuous glucose monitoring (CGM), sensor-augmented insulin pumps, hybrid closed-loop systems, and algorithm-driven decision support tools, have revolutionized the way clinicians and families approach glycemic control [1]. These innovations have not only improved metabolic outcomes but also empowered children and adolescents to participate more actively in their care, enhancing quality of life and reducing diabetes-related distress [2,3,4].

Despite these advances, glucose control remains suboptimal for many young people living with T1D. International data show that a substantial proportion of children and adolescents fail to meet the recommended targets for glycated hemoglobin (HbA1c) and time in range (TIR), particularly during adolescence [5,6]. Disparities persist across socioeconomic, geographic, and ethnic groups, reflecting barriers in access to care, technology, and education [7]. Furthermore, the psychosocial burden of T1D is often underestimated, and critical aspects such as sleep quality, dermatologic complications, and lifestyle behaviors continue to receive limited attention in routine clinical practice [8,9,10].

This Special Issue, “Glycemic Control in Children and Adolescents with Type 1 Diabetes”, brings together five diverse and complementary contributions that help illuminate both the progress made and the challenges that remain. These studies offer insights not only into what is working but also what needs improvement as we strive toward more equitable and effective diabetes care for the next generation.

The five contributions selected for this collection offer a multidimensional view of diabetes management, addressing psychological, technological, clinical, behavioral, and metabolic aspects.

The narrative review by Bombaci et al. explores the psychological and clinical challenges of managing T1D during adolescence, a developmental stage marked by emotional volatility, evolving autonomy, and risk-taking behaviors. The authors remark on the importance of integrating psychological assessment and support into routine diabetes care, highlighting the impact of family dynamics, peer relationships, and mental health on treatment adherence and glycemic variability. Their review also emphasizes the critical role of communication between healthcare providers and adolescents, advocating for a patient-centered approach that builds trust and self-efficacy.

Ceran et al. investigated the impact of dietary habits and sleep deprivation on glucose control in school-aged children with T1D. By employing a cross-sectional design, they found significant associations between irregular meal timing, low-quality diets, insufficient sleep, and suboptimal glucose regulation. These findings reinforce the need for multidisciplinary interventions that address lifestyle factors often overlooked in standard care.

Technological innovation and optimization are central to the contribution of Lejk et al., who compared the metabolic outcomes of children and adolescents treated with different insulin pump systems, ranging from older models without predictive low-glucose suspension to advanced hybrid closed-loop systems. Their data demonstrated better glycemic outcomes, including higher TIR and lower glycemic variability, among users of the most advanced systems, reinforcing the value of technological evolution and integration between CGM and insulin delivery systems.

Ledwoń et al. addressed an often-overlooked dimension of technology use: dermatological complications. In this observational study, nearly all youth using insulin pumps or CGM systems for over three years experienced skin reactions, ranging from scars and lipohypertrophy to eczema and wounds. Notably, these complications were not associated with glycemic outcomes but increased with device use duration. The authors call for enhanced skin care protocols, patient education, and device innovation to reduce these burdens and ensure long-term usability.

Finally, Foti Randazzese et al. examined the discordance between the Glucose Management Indicator and HbA1c in a real-world pediatric cohort. Their results show that relying solely on HbA1c may misrepresent actual glucose control, especially in individuals with significant glycemic variability. This study emphasizes the growing relevance of CGM metrics in both clinical trials and everyday care, urging a shift toward more comprehensive and dynamic evaluation tools.

Looking ahead, the contributions in this Special Issue point toward several critical priorities for clinical care and research. Advanced diabetes technologies, particularly insulin pumps and CGM systems, continue to demonstrate clear superiority over conventional therapies and should be regarded as the standard of care in pediatric diabetes [11,12]. Ensuring universal access to these tools, regardless of geography, socioeconomic status, or healthcare system, is a moral and medical imperative [13].

Adolescence represents a uniquely vulnerable period for individuals with T1D, characterized by fluctuating glucose control, an increased risk of acute complications, and psychological fragility [14]. This age group requires tailored strategies, including specialized education, behavioral support, and therapeutic approaches that acknowledge their specific developmental challenges.

There is a growing awareness of the need to assess and address overlooked aspects of diabetes management. Skin reactions, sleep deprivation, and dietary patterns are not secondary concerns; they are integral to achieving sustainable glycemic control and should be incorporated into both clinical practice and future research frameworks.

We are confident that the insights shared in this Special Issue will contribute to shaping more holistic, equitable, and effective care for children and adolescents living with T1D.

## Abbreviations

The following abbreviations are used in this manuscript:

CGM	Continuous Glucose Monitoring
HbA1c	Glycated hemoglobin
T1D	Type 1 diabetes
TIR	Time in Range
